# The role of contrast-enhanced ultrasound in the diagnosis of hepatic alveolar echinococcosis

**DOI:** 10.1097/MD.0000000000014325

**Published:** 2019-02-01

**Authors:** Diming Cai, Yongzhong Li, Yong Jiang, Huiyao Wang, Xiaoling Wang, Bin Song

**Affiliations:** a Department of Ultrasound; b Department of Pathology; c Department of Psychiatrics; d Department of Operation Management; e Department of Radiology, West China Hospital, Sichuan University, Chengdu, Sichuan Province, China.

**Keywords:** contrast-enhanced ultrasound, hepatic alveolar echinococcosis, liver ultrasound

## Abstract

To evaluate the value of contrast-enhanced ultrasound (CEUS) compared with ultrasound (US) in the diagnosis of hepatic alveolar echinococcosis (AE).

Thirty-one patients with 43 hepatic AE lesions between January 2010 and September 2017 were included in the study. All lesions which were histopathologically proven to be hepatic AE were retrospectively reviewed. Features of the lesions by CEUS were retrospectively studied.

All lesions were detected by US and CEUS in the 31 patients (17 males and 14 females) with a mean age of 38.5 ± 10.6 years (range: 16–58 years). The size of the lesions ranged from 1.5 × 0.7 cm to 15 × 18 cm. By US, 3 lesions (7%, 3/43) were hypoechoic nodules, 21 (48.8%, 21/43) were hyperechoic, and 19 lesions (44.2%, 19/43) were of mixed echogenicity type (solid-cystic). 27 lesions (62.8%, 27/43) had calcifications. Only 1 lesion was detected blood-flow signals. With CEUS, 23 lesions (53.5%, 23/43) displayed no enhancement in the arterial phase, portal phase and delayed phase on CEUS. 11 lesions (25.6%, 11/43) displayed a slight ring-like hyper-enhancement in the arterial phase and displayed hypo-enhancement in the portal and delayed phase. 6 lesions (14%, 6/43) displayed hyper-enhancement in the arterial phase and hypo-enhancement in the portal and delayed phase. 2 lesions (4.7%, 2/43) showed iso-enhancement in the arterial, portal, and delayed phase. 1 lesion (2.3%, 1/43) showed slight hypo-enhancement in the arterial, portal, and delayed phase.

CEUS is a more valid technique for diagnosing AE than US. It could be a reliable tool in the diagnosis of hepatic AE.

## 1 Introduction

Human alveolar echinococcosis (AE), which is caused by the metacestode of the fox tapeworm *Echinococcus multilocularis*, is a near-cosmopolitan zoonosis in the northern hemisphere.^[1,2]^ AE is an important zoonotic tropical disease in China and affects people living in western endemic areas. According to the World Health Organization, AE is highly endemic in western China.^[3,4]^ The disease occurs primarily in the liver and has a profile mimicking slow-growing malignant tumors. AE infection is fatal if left untreated. However, the initial symptoms of AE are usually vague.^[5]^ Many patients have been found to have hepatic lesions by accident and were unable to undergo surgery in the terminal stage, due to delayed diagnosis. Contrast-enhanced ultrasound (CEUS) is a new tool used for the diagnosis of hepatic nodules. CEUS has overcome the limitations of US and can visualize the parenchymal microvasculature. CEUS provides more information for diagnosis of hepatic nodules than US.^[6]^ In the present study, we evaluated the value of contrast-enhanced ultrasound (CEUS) in the diagnosis of hepatic AE.

## 2 Materials and methods

### 2.1 Patient data

We retrospectively reviewed the results of US and CEUS examinations in 31 patients with 43 hepatic AE lesions who were admitted to our hospital between January 2010 and September 2017. All the patients in this study were inpatients at West China Hospital and from Sichuan and Qinghai provinces, China, which are endemic areas consisting of Tibetan communities. All patients underwent serological examinations of AE before surgery; however, only 13 patients were positive. This study was approved by the Ethics Committee of our hospital. All patients underwent surgery, and the diagnoses were confirmed histologically.

### 2.2 US examination

The US and CEUS examinations were performed using a Philips IU22 scanner (Philips Medical Solutions; Mountain View, CA) with a 1 to 5 MHz convex transducer. The ultrasound (US) systems were equipped with harmonic contrast pulse sequencing apparatus. The contrast agent used was SonoVue (BraccoSpa, Milan, Italy) and the suspension contained stabilized sulfur hexafluoride microbubbles.

All patients were asked to fast for 8 hours before US examination. After the patients underwent US examination, the physicians were substitution. The CEUS was performed after US was completed. The physicians of US and CEUS were blind the results of examinations each other. CEUS was started at a low mechanical index (MI: 0.06). SonoVue suspension (2.4 mL) was administered as a bolus injection via the antecubital vein, followed by a 5 mL flush with saline solution. The target lesion and surrounding liver parenchyma were observed continuously for 6 minutes. As previously described by Albrecht et al,^[6]^ the arterial phase was defined as 7 to 30 seconds after contrast agent injection; the portal phase was defined as 31 to 120 seconds after injection, and the late phase was defined as 121 to 360 seconds after injection. The entire CEUS examination was saved as a dynamic digital video file on the hard disk of the US system and recorded on a digital video recorder.

### 2.3 Image analysis

The location, size, shape, boundary and inner echogenicity of the lesions were observed and recorded by US. The enhanced pattern and enhancement level in the different phases of CEUS imaging of the lesion were reviewed. The degree of enhancement was classified as non-enhancement, hypo-enhancement, iso-enhancement, and hyper-enhancement according to the enhancement level of the lesion compared with that of the surrounding normal liver parenchyma. Contrast enhancement patterns were classified as homogeneous, heterogeneous, and rim enhancement. The 2 pairs of examination's results were recorded.

### 2.4 Statistical analysis

The accuracy of hepatic AE diagnosis by US and CEUS was compared and analyzed using SPSS version 18.0 software (SPSS, Inc., Chicago, IL), and the χ^2^ test. *P* <.05 was considered statistically significant.

## 3 Results

### 3.1 US findings and diagnosis

**Figure 1:**
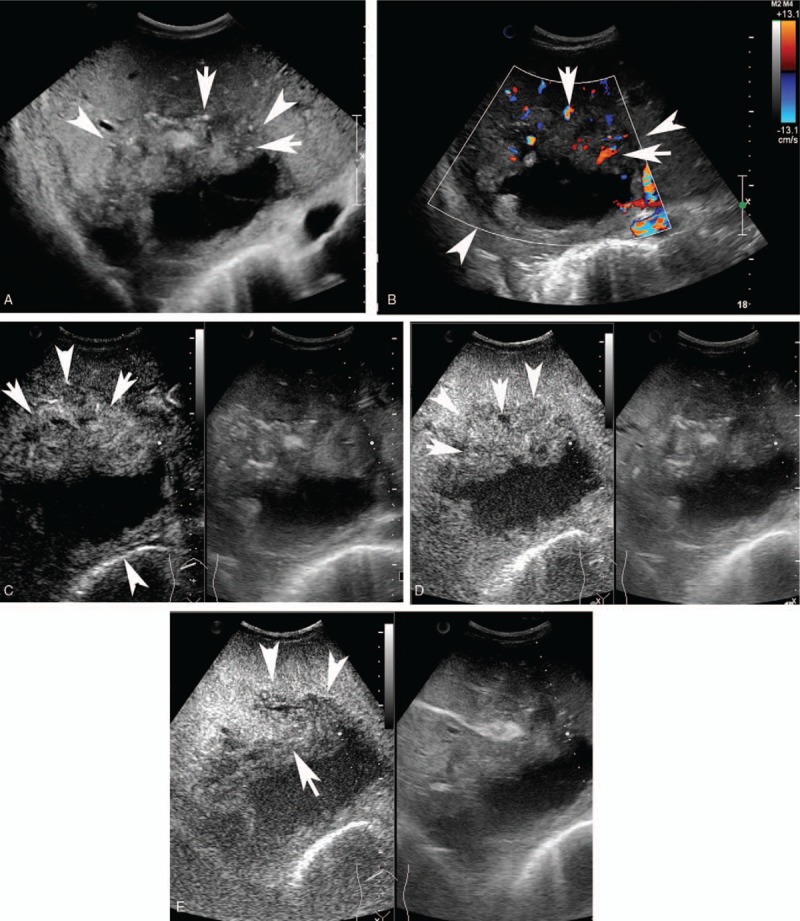
A 29-year-old man with hepatic AE. A: One typical AE lesion (arrow head) with dotted calcifications (arrow) was found in the liver. B: Abundant punctate and linear blood flow signals (arrow) were detected using color Doppler mod in the lesion (arrow head). C: CEUS showed irregular hyper-enhanced (arrow) in the arterial phase in the nodule (arrow head). D and E: In the portal phase and late phase, contrast agent wash-out heterogeneously (arrow) and the un-enhanced area remained. AE = alveolar echinococcosis, CEUS = contrast enhanced ultrasound.

**Figure 2:**
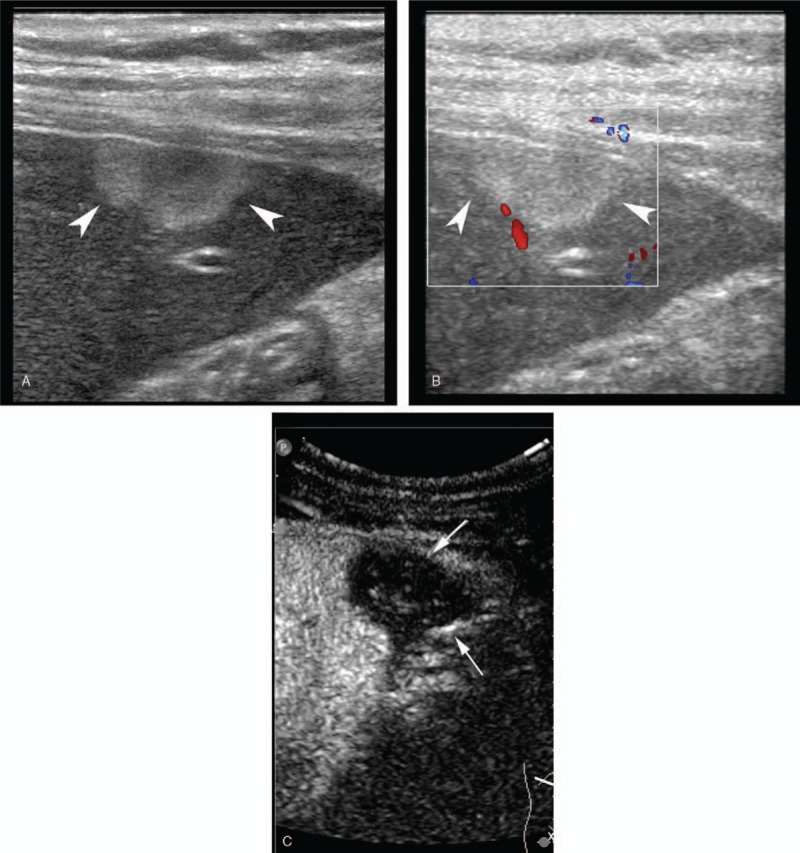
A 40-year-old man with hepatic AE which was misdiagnosed as hepatic hemangioma by US. A: A hyperechoic irregular lesion (arrow head) was found in the left hepatic lobe on US; B: No signal in the lesion (arrow head) was detected using color Doppler mode. C: The lesion (arrow) showed nonhomogeneous hypo-enhancement after contrast agent injection in the late phase by CEUS. CEUS = contrast enhanced ultrasound, US = ultrasound.

All lesions were detected by US. The mean size of the lesions was 7.7 × 6.65 ± 4.7 × 4.9 cm (range from 1.5 × 0.7 cm to 15 × 18 cm). 1 patient had intra-hepatic bile duct dilation, 1 had hydrothorax, 1 had ascites, and 2 had hepatic portal lymph node enlargement. 3 lesions (7%, 3/43) were hypoechoic nodules and 21 (48.8%, 21/43) were hyperechoic nodules. 19 lesions (44.2%, 19/43) were of mixed echogenicity type (solid-cystic). 22 lesions (51.2%, 22/43) had a regular shape and the remainder had an irregular shape. 17 lesions (39.5%, 17/43) had a sharp margin and 26 (60.5%, 26/43) had indistinct margins. 27 lesions (62.8%, 27/43) had calcifications. Only 1 lesion was found blood-flow signals and the rest of lesions were found no blood-flow signals (Figs. 1 and 2).

In this group of patients examined by US, 34 lesions were diagnosed as hepatic AE and 9 lesions were misdiagnosed. (Table 1) The diagnostic accuracy of US was 79% (34/43).

**Table 1:**
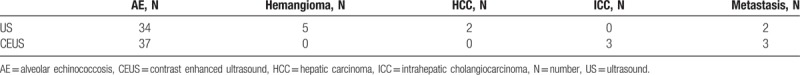
The results of diagnosis in hepatic AE by US and CEUS.

### 3.2 CEUS findings and diagnosis

**Figure 3:**
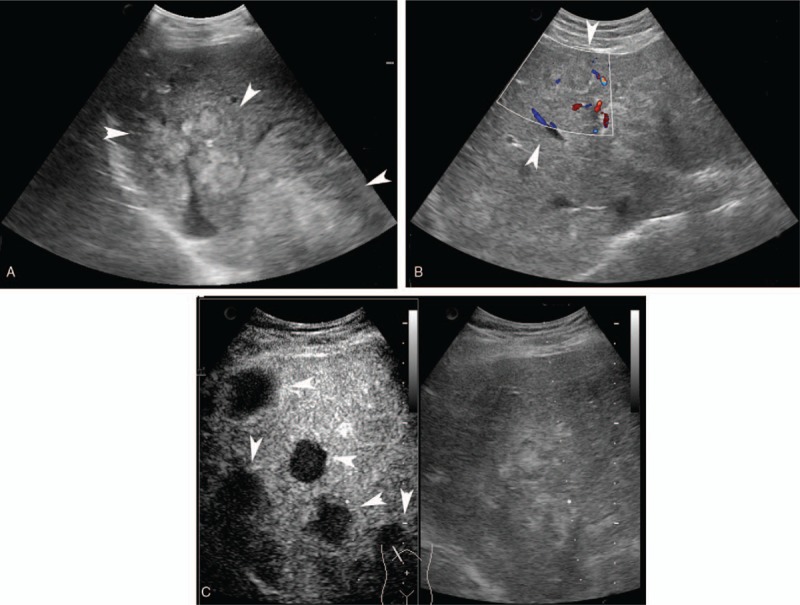
A 48-year-old man with hepatic AE. A: Multiple hyperechoic irregular lesions (arrow head) were found in the right hepatic lobe on US; B: No signal in the lesion (arrow head) was detected using color Doppler mode in the left hepatic lobe. C: CEUS showed multiple lesions (arrow head) with no enhancement in the nodules in the arterial phase which was significantly different from that seen on US. The characteristics of the sonographic image were similar to a “black hole”. AE = alveolar echinococcosis, CEUS = contrast enhanced ultrasound, US = ultrasound.

**Figure 4:**
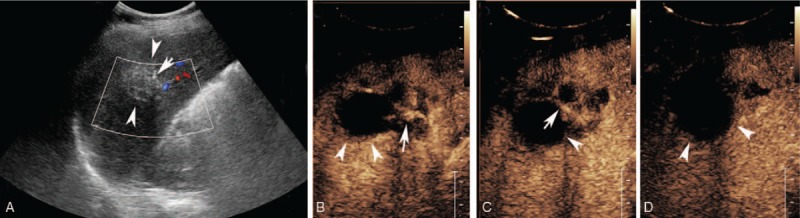
A 46-year-old woman with hepatic AE. A: One nodule (arrow head) with dotted calcifications (arrow) was found in the right hepatic lobe. No signal was detected using color Doppler mode. B: CEUS showed rim enhancement (arrow head) and hyper-enhanced internal septa (arrow) with irregular unenhanced areas in the arterial phase. C and D: In the portal phase and late phase, contrast agent wash-out was seen at the rim (arrow head) and the enhanced septa (arrow), and the un-enhanced area remained. AE = alveolar echinococcosis, CEUS = contrast enhanced ultrasound.

All lesions were detected by CEUS, and 5 forms were determined. 23 lesions displayed no enhancement in the arterial phase, portal phase and delayed phase. 11 lesions displayed slight ring-like hyper-enhancement in the arterial phase and hypo-enhancement in the portal and delayed phase. 6 lesions showed hyper-enhancement in the arterial phase and hypo-enhancement in the portal and delayed phase. 2 lesions displayed iso-enhancement in the arterial, portal, and delayed phase. 1 lesion showed slight hypo-enhancement in the arterial, portal and delayed phase. In 1 patient who had 2 lesions in the liver, the lesions on CEUS were different. Contrast enhancement patterns were heterogeneous with no enhancement in either regular or irregular areas in all lesions (Figs. 3 and 4).

In this group of patients examined by CEUS, 37 lesions were diagnosed as hepatic AE and 6 lesions were misdiagnosed. (Table 1) The diagnostic accuracy of CEUS was 86% (37/43).

### 3.3 Pathological findings

**Figure 5:**
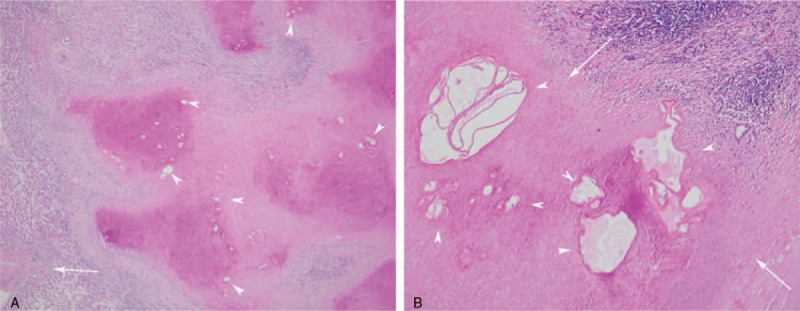
Pathological findings. A: Multiple lesions of shrivelled alveolar hydatid cysts (arrow head) were found microscopically near the parenchyma (arrow), which was strongly positive for eosin (HE staining, 40 × magnification); B: Multiple lesions of shrivelled alveolar hydatid cysts (arrow head) were found and coagulative necrosis within the lesion, surrounded by infiltration of a large number of barrier-like ordered epithelioid cells (arrow) (HE staining, 100 × magnification). HE = hematoxylin and eosin.

Following hematoxylin-eosin (HE) staining, the samples showed a couple of alveolar hydatid cysts which were strongly positive for eosin on microscopic examination. Coagulative or liquefactive necrosis was detected surrounding the cysts. Among the alveolar hydatid cysts, remnant hepatocytes, granulomatous hyperplasia, fibrous tissue, and leucocyte layers were observed (Fig. 5).

### 3.4 Statistical analysis

In the group of patients examined by US, 34 lesions were diagnosed as hepatic AE and 9 lesions were misdiagnosed. And by CEUS, 37 lesions were diagnosed as hepatic AE and 6 lesions were misdiagnosed. The accuracy of hepatic AE diagnosis by US and CEUS was compared and analyzed using SPSS version 18.0 software, and the χ^2^ test. *P* <.05 was considered statistically significant.

CEUS significantly differed from US (χ^2^=6.183, *P* = .0130).

## 4 Discussion

In humans, the larval mass of AE resembles a malignancy in appearance and behavior, as it proliferates indefinitely by exogenous budding and invades the surrounding tissues.^[2,5]^ As AE is asymptomatic in the early stage, it is difficult or impossible to find before it reaches an advanced stage; however, AE has been identified during accidental differentiation of a misdiagnosed malignancy. Patients eventually succumb to hepatic failure, invasion of contiguous structures. The mortality from AE in progressive, clinically manifested cases may be as high as 50% to 75%.^[7]^ Surgery is the first treatment choice for all patients with hepatic AE, and radical resection of the entire hepatic parasitic lesion is the most likely curative procedure.^[8]^ In China and in other endemic areas, due to a lack of medical resources and the atypical features of lesions being ignored by US, most hepatic AE is diagnosed at an advanced stage. This results in increased surgical difficulty and causes many patients to miss the opportunity of efficient treatment, such as surgery.^[9–11]^ In most patients, the prognosis is gloomy with no effective treatment. Thus, early accurate diagnosis of AE is important, especially in endemic areas.

Medical imaging, such as US and computed tomography, has an important role in the diagnosis of hepatic AE. US is accepted as first-choice imaging modalities in the diagnosis and follow-up of hepatic AE. These methods are inexpensive, readily available, and do not involve radiation, and US can be used in endemic regions as a fast diagnostic tool in large populations.^[4,12]^ The typical sonographic appearance of AE includes a mixed heterogeneous echogenic pattern with irregular contours, and cystic necrotic areas or multiple distributed calcified foci.^[13]^ It is regrettable that the features of AE are varied, especially in small lesions.^[14]^ In 2016, Ratzer et al reported that the sonographic appearance of AE could be classified into 6 groups (Ulm classification), including atypical lesions.^[15]^ In our study, 9 lesions were misdiagnosed. We retrospectively analyzed the misdiagnosed lesions and found that the lesions displayed an atypical sonographic appearance of AE. These lesions were hardly to diagnose in AE merely depending on the Ulm classification.

CEUS is a new technique and is useful for evaluating benign and malignant hepatic nodules.^[16–18]^ CEUS has overcome the limitations of gray-scale US, color or power Doppler US, and can visualize the parenchymal microvasculature.^[6]^ Suzuki et al reported that CEUS is beneficial in early diagnosis of the disease.^[19]^ Tao et al described AE lesions as “black holes”.^[20]^ In our study, 12 lesions with bordered areas were identified as “worm-eaten” and 23 lesions were in accordance with those described by Tao S. If the lesions were suspected to be AE by US, CEUS could be chosen to identify their character further. In our study, 6 lesions were misdiagnosed by CEUS. We retrospectively analyzed the reason of misdiagnosed and found that the enhanced mode was accord with some malignancies. In small and atypical lesions, the features of AE by CEUS may include a sharp margin, rim enhancement, and piece-like non-enhanced areas.^[21]^ These features may help to distinguish hepatic AE from malignancy.

We observed that the CEUS features of hepatic AE had diverse forms. Small lesions were in accordance with the report by Cai et al.^[21]^ Large lesions were in accordance with the report by Tao et al.^[20]^ Hepatic AE has been classified into 5 types using MRI by Kodama et al.^[22]^ Maybe, the features of CEUS depend on the stage of AE. In the early stage of AE, the small cysts are interspersed with normal hepatic parenchyma, or the cysts may have internal laminated and germinal layers and are surrounded by residual normal hepatic parenchyma and inflammatory cells that may not all be necrotic. CEUS can display the lesion microcirculation, and the internal components of the lesion should show hypo-enhancement without non-enhancement. These pathological characteristics lead to irregular hypo-enhancement in solid elements and non-enhancement in necrotic or alveolar cysts following injection of contrast agent into the hepatic AE lesions. In advanced AE, the cystic components comprise metacestodal vesicles and liquefactive necrosis; the solid components include coagulation necrosis and calcifications with no or little microcirculation. After injection of contrast agent, CEUS shows non-enhancement, similar to the black holes effect.

We retrospective studied a small number of patients with hepatic AE diagnosed by US and CEUS, and the features of US and patterns of CEUS may not be sufficient to describe the lesions. Further studies are needed to observe the characteristic of CEUS of hepatic AE.

In summary, CEUS is useful method to diagnose the hepatic AE. Patients from endemic areas with hyperechogenicity, mixed echogenicity type, dotted calcification with hypoechogenicity should be suspected of hepatic AE. The atypical performance of nodules by US should receive CEUS examination further. It should be noted that some hepatic AE was accord with enhanced mode of some malignancies and more studied should be performed.

## Acknowledgments

The authors are indebted to Professor Wen-Tiao Wang and Zhe-Yu Chen from the Department of Liver Transplantation Center of West China Hospital, Chengdu, China for providing the cases in this study. We also thank Zheng-Lu Xiong and Lin Lan for their assistance with the injection of contrast agent.

## Author contributions

**Data curation:** Xiao Ling Wang.

**Formal analysis:** Hui Yao Wang.

**Funding acquisition:** Yong Zhong Li.

**Resources:** Yong Jiang.

**Writing – original draft:** Di Ming Cai.

**Writing – review & editing:** Bin Song.

Bin Song orcid: 0000-0003-0484-6583.
